# Non-Invasive Assessment of Lactate Production and Compartmentalization in Renal Cell Carcinomas Using Hyperpolarized ^13^C Pyruvate MRI

**DOI:** 10.3390/cancers10090313

**Published:** 2018-09-05

**Authors:** Renuka Sriram, Jeremy Gordon, Celine Baligand, Fayyaz Ahamed, Justin Delos Santos, Hecong Qin, Robert A. Bok, Daniel B. Vigneron, John Kurhanewicz, Peder E. Z. Larson, Zhen J. Wang

**Affiliations:** Department of Radiology and Biomedical Imaging, University of California San Francisco, San Francisco, CA 94158, USA; Renuka.Sriram@ucsf.edu (R.S.); jeremy.gordon@ucsf.edu (J.G.); celinebaligand@gmail.com (C.B.); fayyaz_ahamed@berkeley.edu (F.A.); justin.delossantos@ucsf.edu (J.D.S.); Hecong.Qin@ucsf.edu (H.Q.); Robert.bok@ucsf.edu (R.A.B.); Dan.vigneron@ucsf.edu (D.B.V.); john.kurhanewicz@ucsf.edu (J.K.);

**Keywords:** hyperpolarized ^13^C magnetic resonance imaging (HP ^13^C MRI), dynamic nuclear polarization (DNP), aerobic glycolysis, lactate dehydrogenase (LDH), lactate efflux, renal cell carcinoma (RCC)

## Abstract

Optimal treatment selection for localized renal tumors is challenging due to their variable biological behavior and limited ability to pre-operatively assess their aggressiveness. We investigated hyperpolarized (HP) ^13^C pyruvate MRI to noninvasively assess tumor lactate production and compartmentalization, which are strongly associated with renal tumor aggressiveness. Orthotopic tumors were created in mice using human renal cell carcinoma (RCC) lines (A498, 786-O, UOK262) with varying expression of lactate dehydrogenase A (*LDHA*) which catalyzes the pyruvate-to-lactate conversion, and varying expression of monocarboxylate transporter 4 (*MCT4*) which mediates lactate export out of the cells. Dynamic HP ^13^C pyruvate MRI showed that the A498 tumors had significantly higher ^13^C pyruvate-to-lactate conversion than the UOK262 and 786-O tumors, corresponding to higher A498 tumor LDHA expression. Additionally, diffusion-weighted HP ^13^C pyruvate MRI showed that the A498 tumors had significantly higher ^13^C lactate apparent diffusion coefficients compared to 786-O tumors, with corresponding higher MCT4 expression, which likely reflects more rapid lactate export in the A498 tumors. Our data demonstrate the feasibility of HP ^13^C pyruvate MRI to inform on tumor lactate production and compartmentalization, and provide the scientific premise for future clinical investigation into the utility of this technique to noninvasively interrogate renal tumor aggressiveness and to guide treatment selection.

## 1. Introduction

The increased utilization of medical imaging has led to a significant increase in the incidental detection of renal tumors, most of which are renal cell carcinomas (RCCs) [1,2]. Many of these incidentally detected RCCs are localized small tumors, which account for 40–50% of newly diagnosed renal tumors [1]. There is increasing recognition of the biological heterogeneity of these tumors [1,3,4], and their management options have evolved over time and now include minimally- and non-invasive approaches, such as ablation and active surveillance, in addition to traditional surgical resection [5]. However, the selection of optimal management approaches for an individual with these renal tumors can be challenging due to a lack of reliable means for estimation of the relative risks and benefits of each approach. This is largely related to the limited ability to pre-operatively predict the histology, grade and aggressiveness of renal tumors. For example, current imaging techniques cannot reliably differentiate low grade, indolent RCCs which are amenable to active surveillance from localized but high grade and potentially aggressive RCCs that require surgery. As a result, many indolent RCCs are likely over-treated with surgery, as the increased detection of RCCs has not translated into a decrease in cancer-specific deaths [1,6]. Additionally, certain benign renal tumors including oncocytomas and lipid-poor angiomyolipomas cannot be reliably differentiated from RCCs by imaging, and are frequently unnecessarily resected [7,8,9]. Renal tumor biopsies also have limitations, including up to 20% of biopsies being non-diagnostic [10,11], and frequent under-grading of RCCs [12,13], which is problematic for selection between active surveillance versus definitive treatment. 

Increasing evidence has shown that RCC is strongly linked to abnormal metabolism. In particular, increased glycolysis with lactate production and efflux, which is thought to promote acid-mediated matrix degradation and tumor invasiveness [14], is a dominant metabolic feature of aggressive RCCs and a potential therapeutic target [15,16,17,18,19]. Lactate dehydrogenase A (*LDHA*) gene encodes the enzyme that catalyzes the conversion of pyruvate to lactate. Multiple studies of RCC tissues have shown that tumor *LDHA* expression correlates with RCC grade and clinical stage, and high tumor *LDHA* is a strong independent predictor of tumor progression and poor patient survival [20,21,22]. To maintain intracellular acid-base balance, cancer cells preferentially utilize the monocarboxylate transporter 4 (MCT4) to export lactate out of the cells. This is essential for preventing a decrease in cytosolic pH, and for maintaining high level of glycolysis and lactate production, thereby supporting cancer cell growth and invasion [23,24]. Prior studies have shown that high MCT4 expression is associated with more aggressive RCCs and worse progression-free survival [25,26]. These studies provide the rationale for metabolic imaging of lactate production and export as a noninvasive means to inform on renal tumor aggressiveness.

Hyperpolarized (HP) ^13^C magnetic resonance imaging (MRI) allows rapid, noninvasive, pathway-specific investigation of real-time metabolic and physiological processes that were previously inaccessible by imaging. Hyperpolarization, achieved through the dynamic nuclear polarization (DNP) technique [27], provides unprecedented gains in sensitivity (>10,000-fold signal increase) for imaging ^13^C-labeled bio-molecules. Dynamic HP ^13^C pyruvate MRI has been used to monitor the LDHA-mediated increased pyruvate-to-lactate conversion that occurs in several aggressive cancers [28,29,30,31]. In addition, recent studies have also combined diffusion-weighted acquisitions with HP ^13^C MRI to interrogate the compartmentalization (i.e., intra- versus extracellular compartment) of ^13^C metabolites including lactate, thereby providing information on the metabolite transporter and microenvironment [32,33,34,35,36,37,38]. In this study, we utilized dynamic HP ^13^C MRI to investigate the pyruvate-to-lactate conversion in a murine orthotopic RCC model, with correlation to tumor *LDHA* expression. Additionally, because rapid lactate export has been associated with more aggressive RCCs, we investigated the lactate compartmentalization via measurements of ^13^C lactate apparent diffusion coefficients (ADCs) by diffusion-weighted HP ^13^C MRI.

## 2. Results

### 2.1. Orthotopic Tumor Characteristics on ^1^H MRI and on Histology

**Figure 1:**
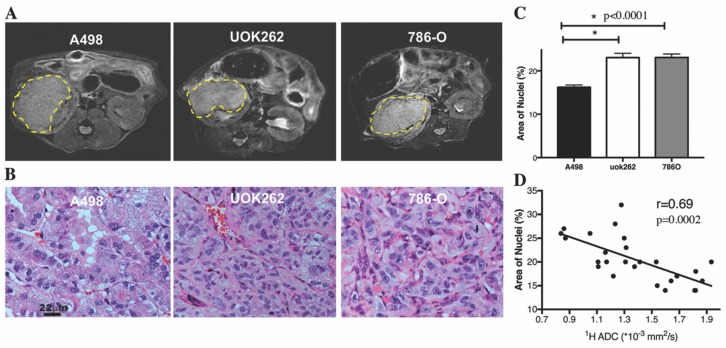
Orthotopic tumor on ^1^H MRI and histology. (**A**) Representative T_2_-weighted anatomic images of the tumors outlined by yellow dashed lines. (**B**) Representative H&E staining of the tumor sections shows qualitatively lower cellularity in the A-498 tumors (at 20× magnification). (**C**) Quantitative image analysis of the H&E sections confirms that the A-498 tumors have significantly lower mean % area covered by nuclei, a measure of cellularity, compared to the other tumors (*p* < 0.0001). The nuclei size is not significantly different among the three tumors. (**D**) The ADCs from ^1^H diffusion-weighted MRI are significantly inversely correlated to tumor cellularity (*r* = 0.69, *p* = 0.0002). * denotes statistically significant change (*p* < 0.05).

The growth characteristics of the orthotopic tumors derived from three human RCC cell lines with varying expression of *LDHA* and *MCT4* is summarized in Supplemental Table S1. The A-498 tumors consistently grew faster (reaching a volume of 0.1 cc under 4 weeks) than the UOK262 and 786-O tumors (reaching a volume of 0.1 cc at ~5.5 weeks). HP ^13^C MRI was performed when the tumor reached a volume >0.4 cc to minimize partial volume averaging with adjacent renal parenchyma. There was no significant difference in tumor volume among the three cell lines at the time of HP ^13^C pyruvate MRI. Figure 1A shows representative T_2_ weighted anatomic images of the orthotopic tumors.

H&E of tumor sections demonstrated that the A-498 tumors were qualitatively less cellular than the UOK262 and 786-O tumors (Figure 1B). Quantitative image analysis of the H&E sections confirmed that the A-498 tumors had significantly lower mean cellularity, represented by the % area covered by nuclei, when compared to the other tumors (A-498: 16 ± 0.5%; 786-O: 23 ± 0.8%; UOK262: 23 ± 1.0%; *p*-values < 0.0001) (Figure 1C). The size of the nuclei was not significantly different among the tumors derived from the three cell lines. As apparent diffusion coefficients (ADCs) from ^1^H diffusion-weighted MRI have been shown to inversely correlate with RCC cellularity in patients [39], we also correlated the ^1^H ADCs of the orthotopic tumors to tumor cellularity as estimated on the H&E sections, and found a significant inverse correlation (*r* = 0.69, *p* = 0.0002) (Figure 1D).

### 2.2. Dynamic HP ^13^C MRI to Interrogate Pyruvate-to-Lactate Conversion

**Figure 2:**
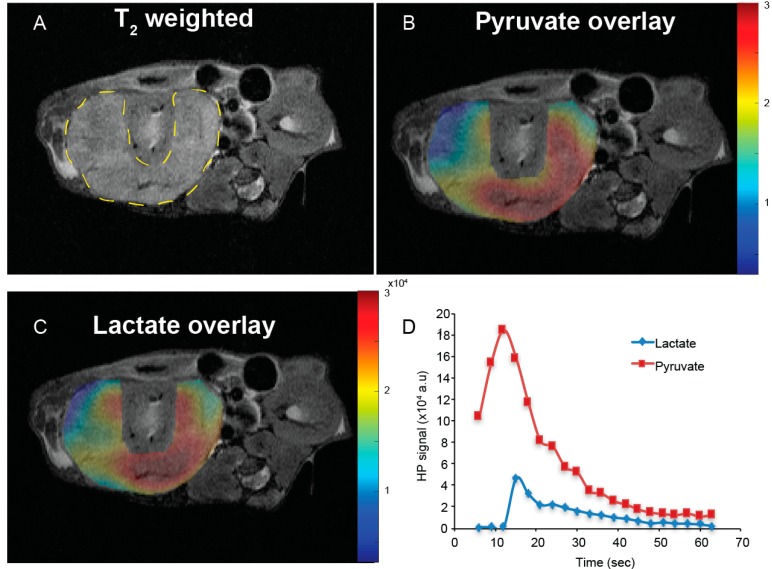
Representative tumor HP ^13^C pyruvate and ^13^C lactate images and dynamic curves of HP ^13^C pyruvate and ^13^C lactate signal over time in an A-498 tumor. (**A**) T_2_-weighted anatomic image with the tumor outlined by dashes. (**B**), (**C**) Tumor HP ^13^C pyruvate and lactate image overlaid on the T_2_-weighted anatomic image at maximum signal intensity at 12 and 15 s, respectively. The ^13^C pyruvate signal in (**B**) is not corrected for the small flip angle. (**D**) Mean tumor signal intensity of HP ^13^C pyruvate and lactate over time. ^13^C pyruvate signal is flip angle corrected. ^13^C lactate signal typically peaks approximately 3–6 s following the peak of the ^13^C pyruvate signal.

**Figure 3:**
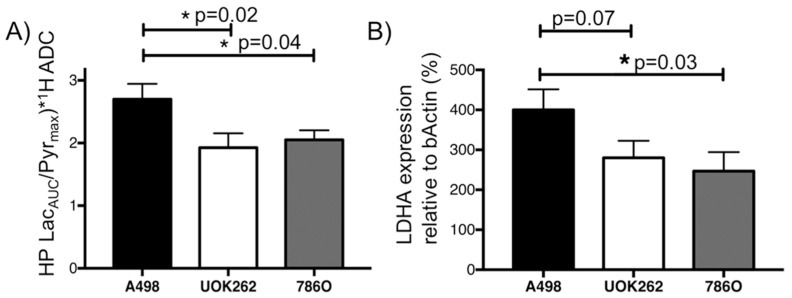
Dynamic HP ^13^C MRI to interrogate pyruvate-to-lactate conversion in orthotopic RCC tumors. (**A**) Tumor ^13^C pyruvate-to-lactate conversion, represented by Lac_AUC_/Pyr_max_, normalized by the tumor ^1^H ADC to adjust for the differential cellularity of the tumors. The normalized pyruvate-to-lactate conversion was significantly higher in the A-498 tumors compared to the UOK262 tumors (*p* = 0.02) and 786-O tumors (*p* = 0.04). (**B**) The *LDHA* expression of A498 tumors was significantly higher than that of 786-O tumors (*p* = 0.03). The *LDHA* expression of A498 was also higher than that of UOK262 tumors, with a p-value approaching significance (*p* = 0.07). * denotes statistically significant change (*p* < 0.05).

Figure 2 shows representative HP ^13^C pyruvate and ^13^C lactate images of tumors overlaid on T_2_-weighted anatomic images, and dynamic curves of HP ^13^C pyruvate and ^13^C lactate signal over time in an A-498 tumor. The ^13^C pyruvate signal peaks around 12 s following the start of the injection. Supplemental Figure S1 summarizes the ^13^C pyruvate dynamics for the tumors derived from the three different cell lines, showing a similar peak ^13^C pyruvate signal among the tumors, and a slightly longer duration of the ^13^C pyruvate signal in the A498 tumors. Supplemental Figure S2 shows representative images of HP ^13^C signal overlaid on T2-weighted anatomic images without tumor segmentation. Figure 3A shows ^13^C pyruvate-to-lactate conversion in A-498 (*n* = 8), UOK262 (*n* = 7), and 786-O (*n* = 8) tumors, represented by the ^13^C lactate area-under-the-curve/maximum pyruvate (^13^C Lac_AUC_/Pyr_max_), and normalized by the tumor ^1^H ADC to adjust for the differential cellularity of the tumors. Since the tumor ^1^H ADC is a surrogate for cell density (Figure 1D), the ^13^C pyruvate-to-lactate conversion in tumors with higher cellularity and corresponding lower ^1^H ADC is scaled to take into account the cellularity, thus permitting a more accurate comparison of relative ^13^C metabolism among the tumors. The tumor HP ^13^C Lac_AUC_/Pyr_max_ ratios without the ^1^H ADC scaling are shown in Supplemental Figure S3. One-way ANOVA with post-hoc comparisons shows that the normalized ^13^C pyruvate-to-lactate conversion was significantly higher in the A-498 tumors (2.70 ± 0.25 × 10^−3^) compared to the UOK262 tumors (1.90 ± 0.23 × 10^−3^, *p* = 0.02) and 786-O tumors (2.05 ± 0.15 × 10^−3^, *p* = 0.04). The tumor *LDHA* expression (relative to β-actin) showed a similar trend with A-498 tumors having significantly higher *LDHA* expression than the 786-O tumors (400 ± 51% vs. 247 ± 47%, *p* = 0.033). The *LDHA* expression of A-498 was also higher than that of UOK262 tumors, with a *p*-value approaching significance (400 ± 51% vs. 280 ± 43%, *p* = 0.071) (Figure 3B).

### 2.3. Diffusion Weighted HP ^13^C MRI to Interrogate Lactate Compartmentalization

**Figure 4:**
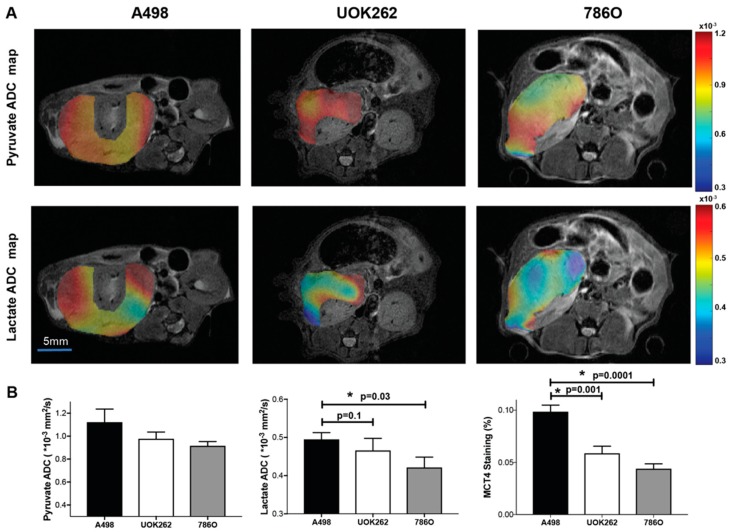
Diffusion-weighted HP ^13^C MRI to interrogate ^13^C lactate compartmentalization in orthotopic RCC tumors (A-498 (*n* = 8), UOK262 (*n* = 7), and 786-O (*n* = 8) tumors). (**A**) Representative ^13^C pyruvate and ^13^C lactate ADC maps of tumors from diffusion-weighted HP ^13^C MRI overlaid on the T_2_-weighted anatomic images. (**B**) The ^13^C lactate ADC values were significantly higher in the A-498 compared to the 786-O tumors (*p* = 0.03). The ^13^C lactate ADC values were also higher in the A-498 tumors compared to the UOK262 tumors, though the p-value did not reach statistical significance (*p* = 0.10). The ^13^C pyruvate ADC values were not significantly different between any of the tumors. MCT4 staining in the A-498 tumors was significantly higher compared to the UOK262 and 786-O tumors (*p* = 0.001, *p* = 0.0001, respectively). * denotes statistically significant change (*p* < 0.05).

Figure 4A shows representative tumor ^13^C pyruvate and ^13^C lactate ADC maps from diffusion-weighted HP ^13^C MRI overlaid on the T_2_-weighted anatomic images. One-way ANOVA with post-hoc comparisons shows that ^13^C lactate ADC values were significantly higher in the A-498 tumors compared to the 786-O tumors (0.495 ± 0.018 × 10^−3^ vs. 0.421 ± 0.027 × 10^−3^ mm^2^/s, *p* = 0.03). The ^13^C lactate ADC values were also higher in the A-498 tumors compared to the UOK262 tumors, though the p-value did not reach statistical significance (0.495 ± 0.018 × 10^−3^ vs. 0.443 ± 0.024 × 10^−3^ mm^2^/s, *p* = 0.10) (Figure 4B). The ^13^C pyruvate ADC values were approximately 2-fold higher than the lactate ADC values, and there was no significant difference in the ^13^C pyruvate ADC values among the three types of tumors studied (A-498: 1.120 ± 0.115 × 10^−3^ mm^2^/s; UOK262: 0.977 ± 0.058 × 10^−3^ mm^2^/s; 786-O: 0.916 ± 0.037 × 10^−3^ mm^2^/s, *p* value > 0.20) (Figure 4B). MCT4 is the key exporter of lactate out of the cells; the MCT4 staining intensity in the A-498 tumors (9.87 ± 0.63%) was significantly higher compared to the UOK262 (5.87 ± 0.68%, *p* = 0.001) and 786-O tumors (4.4 ± 0.47%, *p* = 0.0001) (Figure 4B).

There was no significant correlation between tumor ^13^C lactate ADCs and normalized pyruvate-to-lactate conversion (*r* = 0.288, *p* = 0.32).

## 3. Discussion

Localized small renal cell carcinomas (RCCs) are increasingly detected incidentally at imaging. There is an unmet clinical need for new biomarkers to improve the noninvasive determination of tumor aggressiveness in order to guide treatment selection. In a murine orthotopic model of human RCC, we demonstrated the feasibility of HP ^13^C pyruvate MRI as a noninvasive means to inform on tumor lactate production and compartmentalization, which are strongly associated with aggressive RCCs.

We found that the A-498 tumors had significantly higher ^13^C pyruvate-to-lactate conversion than the UOK262 and 786-O tumors, with a corresponding trend in the tumor *LDHA* expression. We noted that while the tumor ^13^C pyruvate-to-lactate conversion was significantly different between the A-498 and UOK262 tumors, the corresponding difference in the *LDHA* expression between the two tumors did not reach statistical difference (*p* = 0.07). As the tumor ^13^C pyruvate-to-lactate conversion was measured over a much larger tumor volume compared to the *LDHA* expression that was assayed from a very small sample of the harvested tumors; it is possible that any potential intra-tumoral heterogeneity might explain the discrepancy. Nonetheless, these data demonstrate the feasibility of dynamic HP ^13^C MRI to noninvasively inform on tumor *LDHA* expression. This is clinically relevant because multiple prior tissue studies have reported the association between LDHA-mediated lactate production and tumor aggressiveness in RCCs. For example, proteomic and metabolomics analyses of human RCC tissues have shown that lactate was increased in a grade-dependent manner, with high grade RCCs having 2-fold higher lactate than low grade ones [19]. Other studies have demonstrated that RCC LDHA mRNA and protein expression are positively correlated with histological grade and clinical stage, and high LDHA expression is a strong independent predictor of tumor progression and poor survival [20,21,22]. Therefore, by monitoring in vivo the LDHA-mediated pyruvate-to-lactate conversion, HP ^13^C pyruvate MRI may improve the noninvasive stratification of RCC grade and aggressiveness.

We found that the orthotopic tumors derived from the A-498, UOK262 and 786-O cells have different cellularity, with the A-498 tumors having significantly lower cellularity compared to the other two tumors. We also found a significant inverse correlation between the tumor ^1^H ADC from ^1^H diffusion-weighted MRI and tumor cellularity as estimated from the H&E sections. While ^1^H ADC can be influenced by a number of other factors including cell membrane integrity and viscosity, our data are in agreement with those from prior clinical studies of ^1^H diffusion-weighted MRI in RCCs [39], and support the use of ^1^H ADC for in vivo assessment of cellularity. For tumors with higher cellularity per given volume of tumor, the observed hyperpolarized ^13^C pyruvate-to-lactate conversion is expected to reflect both the cellular metabolism (i.e., LDHA mediated pyruvate to lactate conversion) as well as cellularity. We therefore normalized the tumor ^13^C pyruvate-to-lactate conversion by multiplying by the tumor ^1^H ADCs to adjust for the cellularity difference between the tumors in our study. Notably, diffusion-weighted ^1^H MRI is routinely done in the clinic, and diffusion-weighted ^1^H MRI and HP ^13^C MRI can be readily integrated into a multi-parametric MRI exam. Our current data provide the motivation for future work to validate and further refine such an approach incorporating cellularity in the assessment of tissue metabolism with the goal of a more accurate interpretation and comparison of metabolism in tumors with variable cellularity. 

In our study, we used maximum pyruvate (Pyr_max_) to normalize any variability in the ^13^C pyruvate polarization and delivery to the tumors, analogous to the use of maximum standardized uptake value (SUV) in positron emission tomography (PET) to remove the variability in the injected dose. We did not use pyruvate area-under-the-curve (Pyr_AUC_) for normalization, as Pyr_AUC_ will likely be impacted by tumor cellularity. Because pyruvate is rapidly converted to lactate inside the cells, the observed HP ^13^C pyruvate signal is presumed to be predominantly in the extracellular space. As such, we hypothesize that less cellular tumors, such as the A-498 tumors in our study, have larger extracellular space and retain more ^13^C pyruvate signal during the course of the dynamic acquisition, thereby impacting Pyr_AUC_. This is supported by the longer duration of the ^13^C pyruvate signal in the A498 tumors (Supplementary Figure S1). 

In addition to interrogating the tumor HP ^13^C pyruvate-to-lactate conversion, we also investigated the ^13^C lactate compartmentalization using diffusion-weighted ^13^C MRI. Recent work has shown that ADCs of HP ^13^C metabolites can provide information about the localization of these molecules in the tissue microenvironment, with intra-cellular metabolites having lower ^13^C ADCs than the extra-cellular metabolites [33,34]. Relevant to RCCs, a prior in vitro study has reported that cells from an aggressive RCC rapidly exported more lactate out of the cells via the monocarboxylate transporter MCT4 compared to cells from an indolent RCC [28]. Additionally, increased lactate efflux was also observed in RCCs compared to benign renal tumors in an ex vivo HP ^13^C MR study of patient-derived renal tumor tissue slices [40]. Therefore, ^13^C lactate ADCs derived from diffusion-weighted HP ^13^C MRI, by informing on the lactate compartmentalization (relative extra- and intra-cellular distribution), may report on MCT4 transporter expression and provide an additional biomarker of tumor aggressiveness. Indeed, another previous in vitro study of RCC cells demonstrated the ability of diffusion-weighted ^13^C MR to assess in real-time the intra- and extra-cellular ^13^C lactate pools and to probe membrane transport via MCT4 [34]. In that study, the intracellular ^13^C lactate ADCs for RCC cells ranged from 0.17 ± 0.03 × 10^−3^ mm^2^/s to 0.19 ± 0.03 × 10^−3^ mm^2^/s, while the extracellular ^13^C lactate ADCs ranged from 0.57 ± 0.10 × 10^−3^ mm^2^/s to 0.63 ± 0.06 × 10^−3^ mm^2^/s. In our current study, we found that the RCC tumor in vivo ^13^C lactate ADCs ranged from 0.421 ± 0.027 × 10^−3^ mm^2^/s to 0.495 ± 0.018 × 10^−3^ mm^2^/s. These in vivo ^13^C lactate ADCs are between the previously reported intra-cellular and extra-cellular ^13^C lactate ADCs in cell studies, and reflect a composite of intra- and extra-cellular lactate ADCs. Differences in the tumor in vivo ^13^C lactate ADCs can therefore inform on the relative intra- vs. extra-cellular compartmentalization of lactate. We found that the ^13^C lactate ADCs were significantly higher in the A-498 tumors compared to the 786-O tumors. As A-498 tumors have higher MCT4 as quantified by antibody staining, they likely have more rapid ^13^C lactate export to the extracellular compartment, and therefore higher ^13^C lactate ADCs. The ^13^C lactate ADC values were also higher in the A-498 tumors compared to the UOK262 tumors, though the p-value did not reach statistical significance (*p* = 0.10). This may be related to the limited sensitivity of measuring ^13^C lactate ADC at a single time point, and could be improved by dynamic ^13^C ADC measurements [34,37] that aim to monitor the real-time changes in ^13^C lactate compartmentalization due to lactate efflux.

It is possible that the lower cellularity of the A-498 tumors may also contribute to their higher ^13^C lactate ADCs, though the cellularity difference alone is not likely to account for the differences in the observed tumor ^13^C lactate ADCs. The observed ^13^C pyruvate signal is assumed to be predominantly in the extracellular space, which is supported by the observed tumor in vivo ^13^C pyruvate ADCs that were similar to extracellular ^13^C pyruvate ADCs of 1.2 × 10^−3^ mm^2^/s measured in a cell culture system [34]. As such, the ^13^C pyruvate ADCs are likely similarly influenced by cellularity. However, we did not find significant differences in the ^13^C pyruvate ADC values among the three tumors, indicating that the observed ^13^C lactate ADC differences between the tumors would not be explained by the cellularity differences alone. The relative contribution to tumor ^13^C lactate ADC in vivo from lactate export and cellularity needs to be evaluated in future studies. Nonetheless, our results suggest that ^13^C lactate ADCs may provide useful information on tumor lactate compartmentalization. In this study, we did not find a significant correlation between the tumor ^13^C pyruvate-to-lactate conversion and ^13^C lactate ADC values, suggesting that they may provide non-redundant information in vivo. Future studies are needed to evaluate the utility of ^13^C lactate ADCs, alone or in combination with pyruvate-to-lactate conversion in stratifying renal tumor grade and aggressiveness.

Novel imaging biomarkers that can reliably diagnose RCCs and differentiate low from high grade RCCs are of great clinical interest given the rising incidence of incidentally discovered renal tumors and an increasing array of more conservative treatment options [5]. Neither tumor size nor conventional imaging features are able to reliably predict the biological behavior of localized small renal tumors [9,41,42]. Accurate assessment of RCC grade via biopsy is also challenging due to potential intra-tumor heterogeneity [43], and the definition of the Fuhrman nuclear grade of RCC as the highest grade within the tumor even if it only represents a focal region of the tumor. These factors likely accounted for the reported under-grading of RCCs using percutaneous biopsies [12,13]. Given that elevated glycolysis with increased lactate production and efflux is central to RCC malignant behavior, HP ^13^C pyruvate metabolic MRI has the potential to improve the identification of potentially aggressive tumors. Our data from the orthotopic RCC model demonstrate the feasibility of the technique to noninvasively inform on tumor lactate production and compartmentalization, and support its clinical translation for assessing renal tumor aggressiveness and for guiding treatment selection. Notably, the safety and feasibility of HP ^13^C pyruvate MRI has already been demonstrated in the phase I clinical trial in prostate cancer patients [44], and in initial human cardiac study [45]. Additionally, work is ongoing to develop multi-channel ^13^C coil to provide optimal signal reception in abdominal organs for clinical studies.

Our study has several limitations. One limitation is that the tumors generated in this study do not per se represent low versus high grade human RCCs. In our experience, the cell lines derived from indolent human RCCs do not produce robust tumors in vivo in mouse models. Additionally, cell lines of benign human renal tumors are not available. Nonetheless, by investigating the in vivo metabolism of tumors created from human RCC cell lines of differential LDHA and MCT4 expression, which are implicated in the biological behavior of RCCs, we have provided initial data indicating the potential utility of HP ^13^C pyruvate MRI for stratifying tumor aggressiveness and the rationale for future clinical investigation. As already discussed above, another limitation of the study is that the observed in vivo tumor ^13^C lactate ADC is a composite of the intra- and extra-cellular lactate ADC, and may be influenced by both lactate export via MCT4 as well as tumor cellularity. Further work to acquire dynamic ^13^C ADC measurements and to improve the modeling are needed to estimate the tumor intracellular and extracellular lactate fractions, and to refine the interpretation of the observed in vivo tumor ^13^C lactate ADC and its relationship to MCT4. Furthermore, it currently requires two separate ^13^C pyruvate injections to acquire data for measurements of ^13^C pyruvate-to-lactate conversion and ^13^C lactate ADCs. Work is ongoing to combine rapid dynamic imaging for pyruvate-to-lactate conversion measurements with a motion-sensitizing gradient for the simultaneous investigation of lactate compartmentalization.

## 4. Materials and Methods

### 4.1. Cell Lines

The A-498 and 786-O cell lines were derived from patients with the clear cell subtype of RCCs, and purchased from American Tissue Culture Collection (ATCC, Manassas, VA, USA). The UOK262 cell line was derived from a patient with hereditary leiomyomatosis renal cell carcinoma (HLRCC), which is a papillary RCC subtype and is characterized by mutation of the tricarboxylic acid cycle enzyme fumarate hydratase (FH) [46]. UOK262 cells were a kind gift from Dr. W. Marston Linehan (National Cancer Institute, Bethesda, MD, USA). These cell lines were chosen because our preliminary cell studies showed that they have differential *LDHA* and *MCT4* expression (Supplementary Figure S4). Cells were grown in Dulbeco’s Modified Eagle’s medium (DMEM) with 4.5 g/L glucose. The cells were passaged serially and were used between passages 2–12 and at 60–80% confluency for orthotopic (subrenal) tumor implantation.

### 4.2. Murine Orthotopic RCC Model

All procedures were approved by our Institutional Animal Care and Use Committee on 18 June 2016 (Protocol No: AN138870). Four to six week-old male Rag2/IL2rg double knockout mice (B10;B6-Rag2^tm1Fwa^IL2rg^tm1wjl^) were purchased from Taconic Farms or Jackson Laboratories. The Rag2/IL2rg double knockout mice lack T cells, B cells and natural killer cells, and are a useful model for creating xenograft tumors. We selected the renal subcapsular location for tumor implantation to more closely resemble the native tissue environment of renal tumors in patients. Prior studies have noted that the renal subcapsular location provides a superior environment for growth of tumors derived from human RCC cell lines and for studying their biology [47,48]. The mice were anesthetized using 2% inhalant isoflurane and the human RCC cells described above were implanted under the renal capsule using standard procedures. Briefly, a small (1–2 cm) incision was made in the mid-left flank of the mouse to expose the left kidney. An insulin syringe was inserted under the renal capsule, and approximately 5–6 million RCC cells in phosphate buffered solution were injected. The kidney was then placed back inside the abdominal cavity and the wound was sutured close. The mice were screened for tumor growth using T2-weighted ^1^H MRI weekly. A-498 (*n* = 8), UOK262 (*n* = 7), and 786-O (*n* = 8) tumors underwent HP ^13^C MRI and subsequent tissue analyses. Table S1 shows the average volume of the orthotopic tumors at the time of HP ^13^C MRI.

### 4.3. ^1^H and HP ^13^C MRI

Tumor-bearing mice were imaged on a 14.1 T vertical bore micro-imaging system (Agilent, Palo Alto, CA, USA) equipped with high performance gradients (maximum gradient strength = 100 G/cm, maximum slew-rate = 556 G/cm/ms) and a dual tuned 40 mm diameter volume coil for ^1^H and ^13^C imaging (M2M, Australia). Mice were anesthetized with 1–2% inhalant isoflurane. A tail vein catheter was placed for venous access. High resolution respiratory-gated T_2_-weighted ^1^H images were obtained first for anatomic references using a spin echo sequence and the following parameters: matrix size, 256 × 192; field-of-view, 32 × 32 mm; repetition time, 1.6 s; echo time, 20 ms; slice thickness, 1 mm; number of slices, 16–20. ^1^H diffusion weighted images were also acquired through the kidneys and orthotopic tumors using the following parameters: matrix size, 256 × 192; field of view, 32 × 32 mm; slice thickness, 2 mm; *b*-values of 25, 180, 323, 508 s/mm^2^.

[1-^13^C]pyruvate was polarized as previously reported using a HyperSense polarizer (Oxford Instrument, UK) [28]. Briefly, 24 μL [1-^13^C]pyruvic acid was mixed with GE trityl radical (Oxford Instruments, UK) and 1.5 mM Gd-DOTA and polarized for 1 hour, and then dissolved in isotonic buffer with equivalents of NaOH to yield a pH of 6.5–8. Each mouse then underwent two HP ^13^C MR acquisitions following two separate injections of 300 µL of 80 mM HP [1-^13^C]pyruvate: a 2D dynamic echo-planar imaging (EPI) acquisition to interrogate the pyruvate-to-lactate conversion, and a diffusion-weighted ^13^C acquisition to interrogate lactate compartmentalization. HP [1-^13^C]pyruvate was injected via tail vein in 12–14 s.

The 2D dynamic EPI acquisition [49] was performed with spectrally and spatially selective RF pulses to image HP ^13^C pyruvate and ^13^C lactate sequentially using the following parameters: an 8 mm thick axial slab centering on the orthotopic tumor; repetition time, 3 s; matrix size, 16 × 16; field-of-view, 32 × 32 mm; a constant flip angle of 90° for ^13^C lactate; variable flip angles (from 3–90°) for ^13^C pyruvate to efficiently use the hyperpolarized magnetization in the presence of metabolic conversion [50]; a total of 20 time points with the acquisition initiated at 6 s from the start of the injection. The diffusion-weighted HP ^13^C acquisition was performed with spectrally and spatially selective 30° RF pulse for lactate and pyruvate, with all the lactate *b*-value images being acquired before acquiring the pyruvate *b*-value images, and the following parameters: an 8 mm thick axial slab centering on the orthotopic tumor; matrix size, 16 × 16; field-of-view, 32 × 32 mm; interleaved *b*-values, 50, 300, 600, 1500, 1000 and 2000 s/mm^2^; diffusion gradient duration (δ), 5 ms; gradient separation, Δ = 22.4 ms; and respiratory gating (two *b*-values/breath) to minimize signal loss from bulk motion. The single time-point diffusion-weighted HP ^13^C acquisition was initiated at 15 s from the start of the injection.

### 4.4. MRI Data Analysis

All image processing and analysis were performed using MATLAB (Mathworks, Natick, MA, USA), and IDL based in-house software BRIMAGE. T_2_-weighted images were used to calculate the tumor volumes by drawing regions of interest (ROIs) on all slices covering the tumor. From the ^1^H diffusion-weighted images, ^1^H ADC maps were calculated using mono-exponential fitting ($\frac{Sb}{S_{0}}=e^{\left(-b\times ADC\right)}$), where *S_b_* is the signal intensity at a given *b* value, and *S_0_* the signal intensity for *b* = 0 s/mm^2^. The mean tumor ^1^H ADCs were estimated by ROIs from all tumor bearing slices.

All HP ^13^C data were zero filled to 32 × 32. The 2D dynamic spectral spatial ^13^C data were corrected for RF flip angles, and the area under the curve (AUC) of ^13^C lactate to maximal pyruvate signal (Lac_AUC_/Pyr_max_) was calculated from ROIs drawn over the tumor. To account for the different cellularity between the tumors in vivo, which could impact the comparison of cellular pyruvate metabolism, tumor ^13^C pyruvate-to-lactate conversion was scaled by the tumor ^1^H ADC. ^1^H ADC has been shown previously to inversely correlate with RCC cellularity in patients [39]. As such, for example, the ^13^C pyruvate-to-lactate conversion in tumors with higher cellularity and corresponding lower ^1^H ADC is scaled by multiplying Lac_AUC_/Pyr_max_ by the mean tumor ^1^H ADC to take into account the cellularity.

For the diffusion-weighted ^13^C MR acquisition, the same mono-exponential fitting as above was performed to compute the mean ^13^C lactate and ^13^C pyruvate ADC of the tumors.

### 4.5. LDHA Expression Assay

Following imaging, mice were euthanized and the renal tumors were rapidly dissected. A portion of the tumor was immediately formalin-fixed for hematoxylin and Eosin (H&E) and MCT4 staining. The rest was snap frozen and stored at −80 °C for subsequent LDHA expression assay.

mRNA expression of *LDHA* was quantified using qRT-PCR. In brief, total RNA was extracted from the tumor tissue using an RNAeasy kit (Qiagen, Germantown, MD, USA). Reverse transcription was performed using an iScript cDNA Synthesis kit (BioRad Laboratories, Hercules, CA, USA), and the subsequently generated cDNA was used for PCR in triplicate with TaqMan chemistry on the PikoReal system (Thermo Fisher Scientific, Waltham, MA, USA. Primers for the genes were obtained from Thermo Fisher Scientific (Waltham, MA, USA). β-Actin was used as the reference gene for expression level quantification.

### 4.6. H&E and MCT4 Staining, and Imaging Analysis

Standard H&E as well as anti-MCT4 staining (rabbit, polyclonal IgG, Santa Cruz Biotechnology Inc, USA) were performed on tumor sections (3 μm thick) de-paraffinized overnight by incubation at 55 °C and in descending grade of ethanol using standard protocols. Heat-induced epitope retrieval was performed with citrate buffer at pH 6.0 for 20 min and cooled, followed by endogenous quenching with 30% H_2_O_2_ for 15 min. For MCT4 staining, tissue sections were incubated with rabbit polyclonal primary antibody SC-376140 anti MCT4 in 1:100 dilution at 4 °C overnight, followed by incubation with rabbit secondary antibody/ HRP at 1:200 dilution for 20 min at room temperature. The stained sections were then imaged with a Nikon 6D microscope under bright-field illumination using a 40× power objection yielding a 0.15 µm in-plane resolution.

The H&E images were used to estimate tumor cellularity using a home built CellProfiler pipeline (Figure S5) [51]. Briefly, the hematoxylin stain was first isolated by un-mixing the colors in order to allow the counting of the number of nuclei. The image masks of the nuclei were then created after Otsu thresholding, prescribing the cell diameter, and optimizing the de-clumping of objects. Finally, the cellularity of the tumors was estimated by calculating the percentage area of the image covered by nuclei. All of the images were obtained at the same resolution. The accuracy of the estimated cellularity of the tumor obtained using the method above was also manually verified in a subset of cases.

The MCT4 images were analyzed using the Image analysis toolbox in MATLAB (Figure S6). The HSV (hue-saturation-value) color scale was used to threshold the images and create binary images of the MCT4 stain using an interval of [0, 0.169] for hue (H) to capture the brown color stain, interval of [0.189, 1.000] for saturation (S), and restricting the value (V) to within the 16th percentile to account for the different illumination. Using the masks, the number of stained (MCT4) pixels was calculated as a percent of the total tissue area after sampling three different regions for each tumor, and was represented as the mean % MCT4 staining for each tumor. More detailed methodologies for the image analyses are described in the Supplementary data. 

### 4.7. Statistical Analysis

Data were presented as mean **±** standard error of the mean. One-way ANOVA with post hoc tests were used to assess the difference among relevant groups. Corrections for multiple comparisons were made by controlling for false discovery rate (*q* < 0.05) using the two-stage step-up method of Benjamin, Krieger and Yekutieli. Correlation analyses were performed using Pearson correlation tests. *p*-values < 0.05 were considered statistically significant. All statistical tests were performed using PRISM (GraphPad, La Jolla, CA, USA).

## 5. Conclusions

In summary, we have demonstrated the feasibility of HP ^13^C pyruvate MRI to noninvasively interrogate tumor lactate production and compartmentalization in a murine orthotopic model of human RCCs, and to inform on tumor *LDHA* and MCT4 expression which are implicated in tumor aggressiveness. Our data provide the scientific premise for future clinical investigation into the utility of HP ^13^C pyruvate MRI to noninvasively differentiate benign renal tumors from RCCs and to stratify indolent from aggressive RCCs. 

## Supplementary Materials

The following are available online at http://www.mdpi.com/2072-6694/10/9/313/s1. Figure S1: Hyperpolarized ^13^C pyruvate dynamics in orthotopic tumors; Figure S2: Representative non-segmented images of hyperpolarized ^13^C signal overlaid on T_2_-weighted anatomic images; Figure S3: Tumor HP ^13^C Lac_AUC_/ Pyr_max_ ratios, without ^1^H ADC normalization and ^1^H ADC in the three orthotopic tumors; Figure S4: mRNA expression of RCC cells grown in 2D culture; Figure S5: Assessing cellularity from H&E images using a multi-step CellProfiler pipeline; Figure S6: Image analysis of MCT4 staining using Matlab image analysis toolbox; Table S1: Tumor characteristics.
