# Supplementary material for: Non-Invasive Assessment of Lactate Production and Compartmentalization in Renal Cell Carcinomas Using Hyperpolarized 13C Pyruvate MRI
Source: Cancers (Basel). 2018 Sep 5;10(9):313. doi: 10.3390/cancers10090313 (PMC6162434; doi:10.3390/cancers10090313)
Supplement: Supplementary file 1 [file cancers-10-00313-s001.pdf]

Supplementary Materials: Non-Invasive Assessment of Lactate Production and Compartmentalization in Renal Cell Carcinomas Using Hyperpolarized ^{13}C Pyruvate MRI

Renuka Sriram, Jeremy Gordon, Celine Baligand, Fayyaz Ahamed, Justin Delos Santos, Hecong Qin, Robert Bok, Daniel B. Vigneron, John Kurhanewicz, Peder E. Z. Larson and Zhen J. Wang

Table S1. Tumor characteristics.

Characteristics	A-498	UOK262	786-O
RCC subtype	Clear cell	Papillary	Clear cell
Mean time to grow into 0.1 cc tumor from time of implantation (weeks)	4	5.5	5.5
Average tumor volume at hyperpolarized ^{13}C MRI (cc)	0.43 ± 0.11	0.49 ± 0.07	0.56 ± 0.07

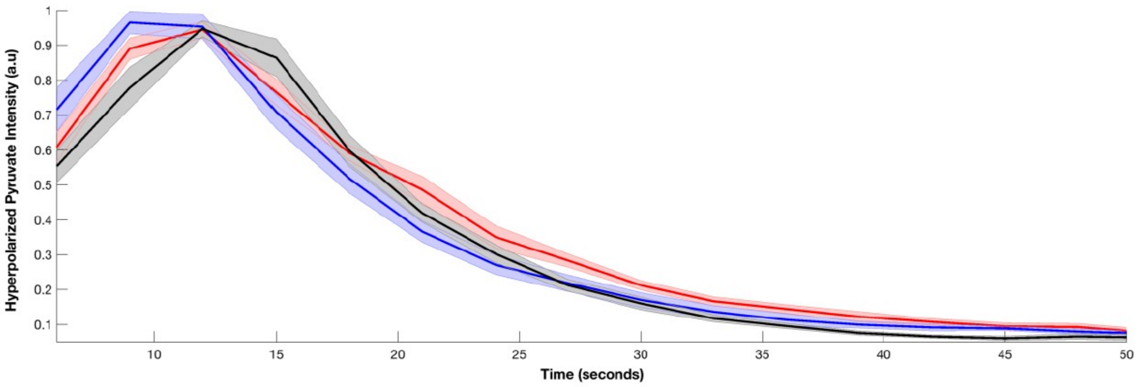

Figure S1. Hyperpolarized ^{13}C pyruvate dynamics in orthotopic tumors. There is similar peak hypeprolarized ^{13}C pyruvate signal across the orthotopic tumors. A-498 tumors (red line) show slightly longer duration of the hyperpolarized 13C pyruvate signal. A-498 (red line), UOK262 (black line), and 786-O cells (blue line). The solid line represents the mean value, and the shaded area represents the standard error.

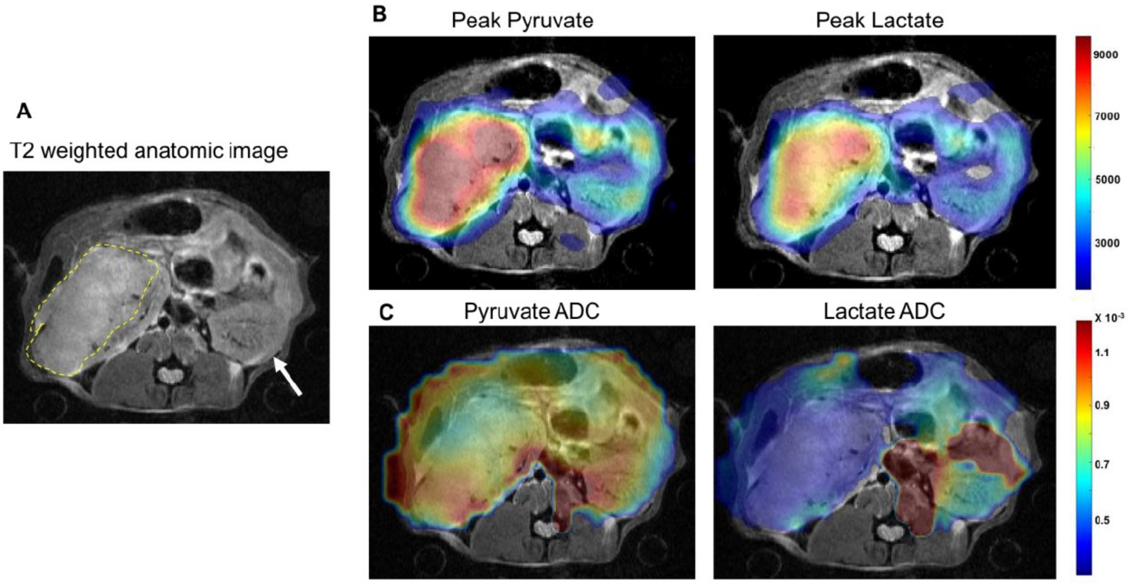

Figure S2. Representative images of hyperpolarized ^{13}C signal overlaid on T_2-weighted proton images from a 786-O tumor bearing mouse. (**A**) T_2-weighted anatomic image showing orthotopic tumor (outlined in yellow) and the contralateral kidney (white arrow). (**B**) Hyperpolarized ^{13}C pyruvate and lactate signal at their peak intensity overlaid on T_2 weighted image. (**C**) Hyperpolarized ^{13}C ADC maps of pyruvate and lactate (scaled by 10^{-3}).

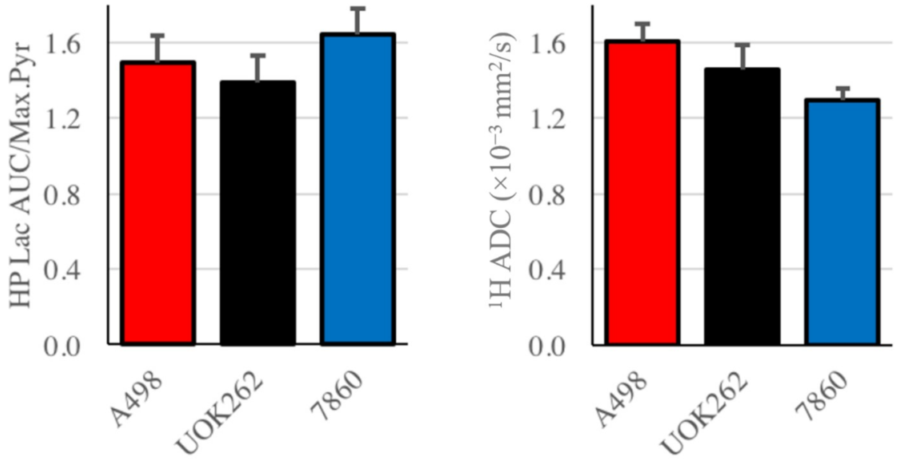

Figure S3. Left: Bar graph of tumor hyperpolarized ^{13}C Lactate area-under-the-curve to maximum pyruvate signal ratio (HP ^{13}C Lac_{AUC}/Pyr_{max}), without ^{1}H ADC normalization. Right: Bar graph of tumor mean ^{1}H ADC.

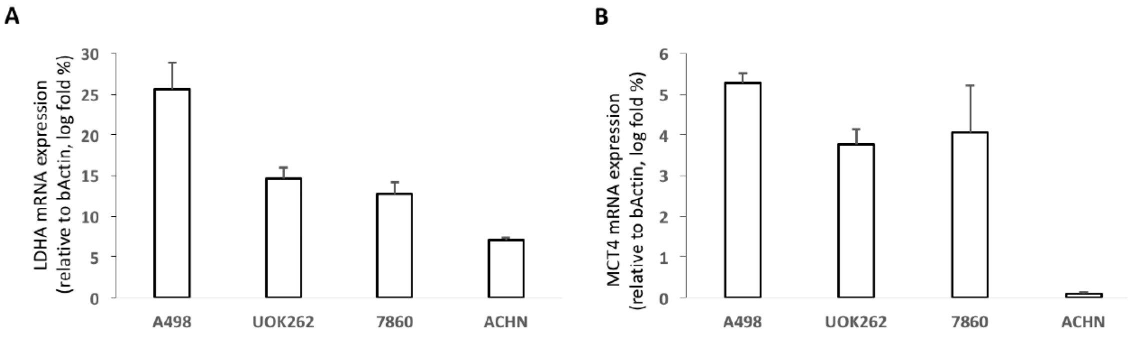

Figure S4. mRNA expression of RCC cells grown in 2D culture (**A**) *LDHA* and (**B**) *MCT4*. We evaluated 4 RCC cell lines for potential in vivo tumor imaging study. A-498 and 786-O are cell lines derived from human clear cell RCCs and demonstrate distinct differences in *LDHA* and *MCT4* expression. UOK262 cell line shows represents a papillary subtype of RCC with similar *LDHA* and *MCT4* mRNA expression compared to the 786-O cell line. In preliminary studies, these 3 cell lines showed consistent growth into solid well perfused tumors in mice, and were subsequently used for metabolic evaluation. ACHN is a cell line that has been used as a model of papillary RCC, and shows distinctly lower expression of *LDHA* and *MCT4*. However, in preliminary studies, the ACHN cells formed cystic tumors that were poorly perfused, and therefore not used for subsequent metabolic investigation.

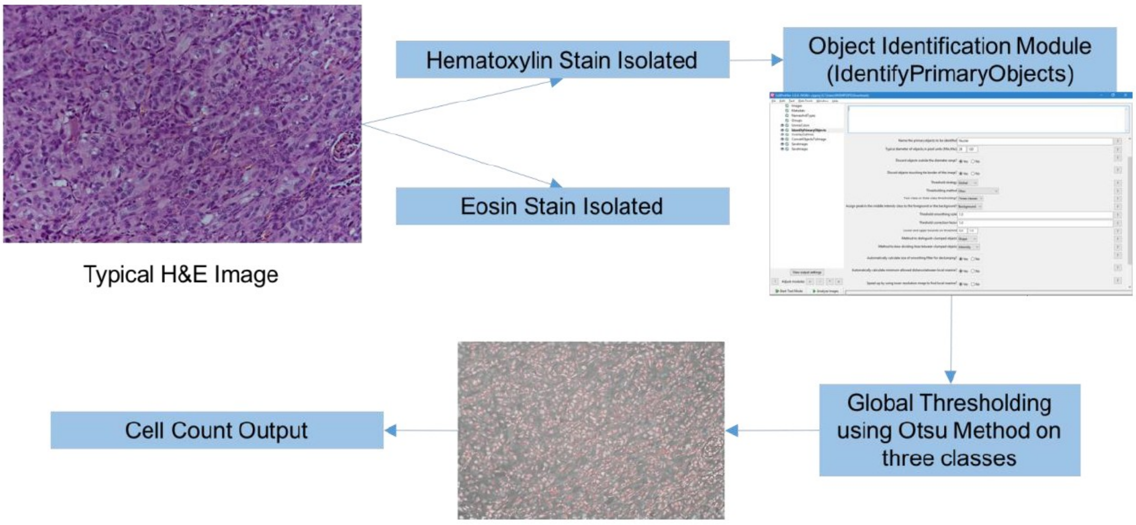

Figure S5. Assessing cellularity from H&E images using a multi-step CellProfiler pipeline. The first step separated the image into a hematoxylin stain image and an eosin stain image using the UnmixColors module. The hematoxylin stain image was used in the following steps to count nuclei. The next step was the IdentifyPrimaryObjects module, which used the Otsu Thresholding method and Global Thresholding strategy to minimize weighted variance in a machine learning algorithm to detect the appropriate pixel intensity that represents a cell's nucleus. Then, depending upon the individual image, in particular the cell size and the contrast between the nuclei and the background, the following parameters were modified to achieve the most accurate cell count: typical diameter of objects, number of threshold classes, automatic or manual thresholding, method of distinguishing clumped objects, and method of drawing dividing lines. Next, an overlay image was generated where the detected nuclei outlines were superimposed on top of the hematoxylin stain image in the "OverlayOutlines" module for visual inspection. The aforementioned parameters were optimized for individual images if necessary, and the process was repeated. To assess the accuracy of nuclei count, random sections of the image were selected and the nuclei were counted manually. If the manual count of the nuclei was greater than 1.5 times of the interquartile range, the image was discarded. A binary image of the nuclei was then made using "ConvertObjectsToImage" module, and used for

estimating the number of nuclei, their diameter (median, 10th percentile, 90th percentile), and finally their area by thresholding the nuclei size after applying de-clumping and smoothing.

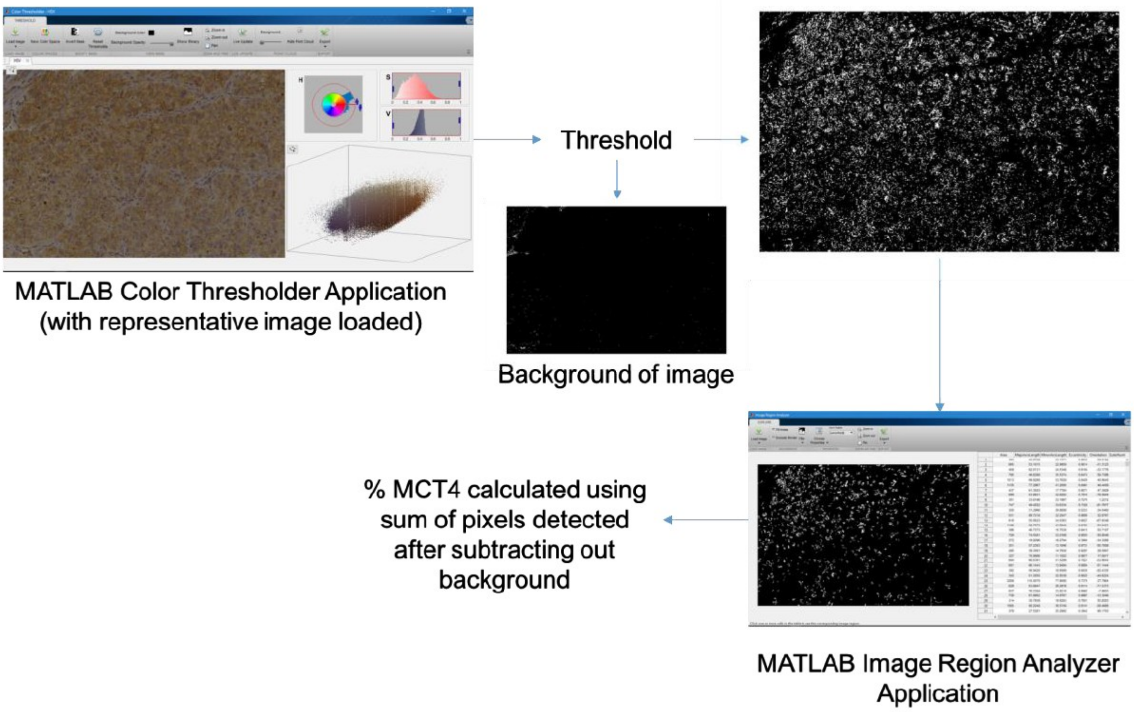

Figure S6. Image analysis of MCT4 staining using Matlab image analysis toolbox. The HSV (hue-saturation-value) color scale was used to threshold the images and create binary images of the MCT4 stain using interval of [0,0.169] for hue (H) to capture the brown color stain, and interval of [0.189,1.000] for saturation (S). Due to differing illumination from image to image, the allowed values (V) were restricted to within 16th percentile to capture only the MCT4 expression on the cell membrane. Next, image regions were analyzed from the resulting binary image, and the pixel count of the detected MCT4 regions was generated. The same protocol was adapted to select the background in every image: hue = [0.113,0.738], saturation = [0,0.161], value = 72th to 100th percentile. The pixel count for the background was then generated. The percentage of MCT4 expression for each tumor section was represented as: MCT4 pixel count/(total pixel count−background pixel count).

© 2018 by the authors. Licensee MDPI, Basel, Switzerland. This article is an open access article distributed under the terms and conditions of the Creative Commons Attribution (CC BY) license (http://creativecommons.org/licenses/by/4.0/).
